# Ginsenoside Rg1 improves bone marrow haematopoietic activity *via* extramedullary haematopoiesis of the spleen

**DOI:** 10.1111/jcmm.12643

**Published:** 2015-07-08

**Authors:** Hua-Hsing Liu, Fei-Peng Chen, Rong-Kai Liu, Chun-Lin Lin, Ko-Tung Chang

**Affiliations:** Department of Biological Science and Technology, National Pingtung University of Science and TechnologyPingtung, Taiwan

**Keywords:** haematopoietic stem cells, bone marrow, extramedullary hematopoiesis, spleen, cyclophosphamide

## Abstract

Cyclophosphamide (CY) is a chemotherapeutic agent used for cancer and immunological diseases. It induces cytotoxicity of bone marrow and causes myelosuppression and extramedullary haematopoiesis (EMH) in treated patients. EMH is characterized with the emergence of multipotent haematopoietic progenitors most likely in the spleen and liver. Previous studies indicated that a Chinese medicine, ginsenoside Rg1, confers a significant effect to elevate the number of lineage (Lin^−^) Sca-1^+^ c-Kit^+^ haematopoietic stem and progenitor cells (HSPCs) and restore the function of bone marrow in CY-treated myelosuppressed mice. However, whether the amelioration of bone marrow by Rg1 accompanies an alleviation of EMH in the spleen was still unknown. In our study, the cellularity and weight of the spleen were significantly reduced after Rg1 treatment in CY-treated mice. Moreover, the number of c-Kit^+^ HSPCs was significantly decreased but not as a result of apoptosis, indicating that Rg1 alleviated EMH of the spleen induced by CY. Unexpectedly, the proliferation activity of c-Kit^+^ HSPCs was only up-regulated in the spleen, but not in the bone marrow, after Rg1 treatment in CY-treated mice. We also found that a fraction of c-Kit^+^/CD45^+^ HSPCs was simultaneously increased in the circulation after Rg1 treatment. Interestingly, the effects of Rg1 on the elevation of HSPCs in bone marrow and in the peripheral blood were suppressed in CY-treated splenectomized mice. These results demonstrated that Rg1 improves myelosuppression induced by CY through its action on the proliferation of HSPCs in EMH of the spleen and migration of HSPCs from the spleen to the bone marrow.

## Introduction

Extramedullary haematopoiesis (EMH) is the production of mature blood cells outside of the medullary cavity of bones 1. It is a normal physiological process during foetal development where primitive haematopoietic stem cells (HSCs) initially emerge in the yolk sac and migrate stepwise into aorta-gonad-mesonephros, placenta, liver and spleen before final lodgement in the bone marrow 2. However, EMH usually occurs as a pathological compensation in adults as a result of bone marrow malfunction in patients 3. Extramedullary haematopoiesis is characterized microscopically by the presence of mixed population of haematopoietic precursor cells including erythroid lineage, myeloid lineage and megakaryocyte 4. In most reported cases, the foci of EMH appear in the spleen and liver of patients but atypical sites can be seen occasionally 5. Extramedullary haematopoiesis usually attracts clinical awareness only while a compressive or obstructive haematopoietic mass appears to threaten the patient's life 6,7.

Cyclophosphamide (CY) is one of the drugs that causes EMH while used in cancer chemotherapy, immunosuppressive therapy and for a conditioning regimen in patients prior to HSC transplantation 8,9. In the laboratory, treatment of CY causes myelosuppression in mice and induces mobilization of haematopoietic progenitors into the peripheral blood followed by the occurrence of EMH in the spleen. An increased frequency of colony-forming units in the spleen (CFU-S) as well as CD34^+^ and CD117^+^ haematopoietic stem and progenitor cells (HSPCs) can be detected after CY administration 10,11. On a biology basis, EMH induced by CY reflects the transition of balances among HSPCs and their original and new microenvironments in the bone marrow and the spleen, respectively. However, the possible benefit underneath this biological phenomenon is so far unknown.

Panaxatriol and Panaxadiol saponins are the main constituents extracted from the root of *Panax ginseng*, a herbal medicine popularly used in Asia for the treatment of cardiovascular diseases, diabetes, cancer and inflammation 12–15. Ginsenoside Rg1, a steroid-like structure with the sugar moieties modification 16, is the most active component of Panaxatriol saponins. Recently, the pharmacological effects of Rg1 has been reported to help restore bone marrow HSPCs and lymphopoiesis in myelosuppressed mice induced by CY 17. The amelioration of bone marrow function might relieve the haematopoietic stress in the spleen caused by CY. Thereby, we have suggested that the treatment of Rg1 in myelosuppressed mice induced by CY not only improves the haematopoietic activity of bone marrow, but also alleviates EMH in the spleen. Therefore, the biological effect of Rg1 on the expansion of HSPCs was further investigated in CY-treated mice.

## Materials and methods

### Mice

Eight-week-old adult female C57BL/6J mice, weighing 20 ± 5 g, were purchased from National Laboratory Animal Center (Taipei, Taiwan) and housed in a clean conventional animal facility at 22°C with 12-hr light/dark cycle. Sterilized food and water were freely accessible in their cage. Mice were then randomly selected for control, CY and CY plus Rg1 groups. Mice in CY and CY plus Rg1 group were administrated with CY [50 mg/kg, intraperitoneal (i.p); Sigma-Aldrich, St. Louis, MO, USA] once a day for 4 days, followed by daily peritoneal injection of equal volume of normal saline or ginsenoside Rg1 (15 mg/kg/day; ChromaDex, Irvine, CA, USA), respectively at 9:00 a.m. in the morning for 7 days. The protocol was approved by the Institutional Animal Care and Use Committee of College of Veterinary Medicine at National Pingtung University of Science and Technology.

### Splenectomy

Mice were randomly assigned into two groups receiving sham operation or splenectomy. The operation was referred by a previous report 18. Briefly, right before operation, mice were anaesthetized with 1.2% Tribromoethanol (0.23 ml/10 g b.w, i.p; Sigma-Aldrich). After skin was shaved and sterilized with 10% povidone-iodine solution (Tien Chen Pharmaceutical Co, Tainan, Taiwan), an ~1 cm incision was made on the left side of the abdominal cavity under the rib cage. The spleen was then removed by cutting the mesentery and connective tissue and the splenic vessels were cauterized. For sham-control mice, incisions were made without removing the spleen. The animals were kept at 37°C until wakeup. After 2 weeks mice with no sign of inflammation in the abdominal cavity were assigned for the next experiment.

### Cyclophosphamide and ginsenoside Rg1 treatment

Mice received i.p injection of CY at the dose of 50 mg/kg once a day for 4 days, followed by daily peritoneal injection of equal volume of normal saline or ginsenoside Rg1 (15 mg/kg/day) for 7 days. The mice were killed 16 hrs after the last dose of Rg1. CY was obtained from Sigma-Aldrich Corporation. Ginsenoside Rg1 (purity ≧95%) was purchased from ChromaDex^®^ Corporation.

### Hematopoietic stem/progenitor cells analysis

Cells from the bone marrow and spleen were collected 16 hrs after the last dose of Rg1 and were counted before use. Bone marrow cells were flushed from pairs of femur and tibia with a 25-gauge needle, and spleen single cell suspension was prepared by dissociating the whole spleen tissue with a glass grinder. Cells were collected in buffer solution containing PBS (J.T Baker, Phillipsburg, NJ, USA) in pH 7.4, with 0.5% bovine serum albumin (BSA) (Sigma-Aldrich). Peripheral blood was collected in ethylenediaminetetraacetic acid (Sigma-Aldrich) by facial jugular vein bleeding. An equal volume (50 μl) of the peripheral blood was added into 3 ml cold lysis buffer for 15 min. at room temperature with occasional and gentle shaking. The peripheral blood cells were then washed twice with PBS/BSA (0.5%). After centrifugation (300 × g) at 4°C for 5 min., the peripheral blood cells were resuspended in 1 ml PBS. The analysis of HSPCs subsets was performed by flow cytometry using biotin-conjugated antimouse lineage antibodies [CD3 (5 μg/ml), B220 (5 μg/ml), Ter119 (2.5 μg/ml), Gr-1 (0.6 μg/ml), CD11b (0.6 μg/ml)-Biotin], fluorescein isothiocyanate (FITC)-conjugated streptavidin (5 μg/ml), phycoerythrin (PE) -conjugated CD117 (c-Kit; 2 μg/ml), Peridinin chlorophyll (PerCP)-conjugated Ly-6A/E (Scal-1; 3 μg/ml). The identification of HSPCs in peripheral blood was performed with FITC-conjugated CD45 (5 μg/ml) and PE-conjugated CD117 (2 μg/ml). All antibodies were obtained from Biolegend (San Diego, CA, USA).

### Detection of apoptosis by Annexin V assay

Apoptosis was analysed in the spleen and bone marrow cells by flow cytometry using the Annexin V/7-amino-actinomycin D (catalog no. 559763; BD Pharmingen, San Diego, CA, USA) staining procedure. Briefly, the cells were washed twice with cold PBS and resuspended in binding buffer and incubated with 5 μl of Annexin V-PE and 5 μl of 7-AAD for 15 min. at room temperature. The cells were then analysed within an hour following manufacturer's protocol from BD Biosciences (San Jose, CA, USA) Annexin V-PE Apoptosis Detection Kit.

### Cell cycle analysis

*In vivo* incorporation of 5-bromo-2-deoxyuridine (BrdU) into splenic and bone marrow cells was assessed by using the FITC-BrdU Flow kit (catalog no. 559619; BD Pharmingen). Mice were intraperitoneally injected with 100 μl of BrdU (10 mg/ml; BrdU) and killed after 2 hrs. The splenic and bone marrow cells were collected and stained with PE-CD117 antibody (2 μg/ml; Biolegend) followed by washing, fixing and permeabilizing with the BD Cytofix/Cytoperm Buffer. After repeated incubation on ice, wash and centrifugation twice, cells were treated with DNase (0.3 mg/ml; catalog no. D-4513) for 1 hr at 37°C to expose BrdU epitope. By staining with 1× FITC-conjugated anti-BrdU antibody for 20 min. at room temperature, the staining buffer containing 7-amino-actinomycin D (7-AAD) was added to each tube, and cells were resuspended and analysed by flow cytometry. The combination of BrdU and 7-AAD permits the enumeration and characterization of cells that are actively synthesizing DNA (BrdU incorporation) in terms of their cell cycle position (*i.e*., G0/1, S, or G2/M phases defined by 7-AAD staining intensities).

### Histological analysis

The fresh femur and spleen were fixed in 10% neutral formalin (Shimakyu's Pure Chemicals, Osaka, Japan) overnight. Femur was decalcified with a mixture solution of sodium citrate (J.T Baker) and formic acid (Sigma-Aldrich) for another 24 hrs before paraffin-embedding (Leica Biosystems, Buffalo Grove, IL, USA). Paraffin sections by 5-μm thickness were prepared for haematoxylin and eosin (Merck & Co., Inc., Whitehouse Station, NJ, USA) staining and the histological review was performed under an Olympus CX41 light microscope (Center Valley, PA, USA).

### Statistical analysis

All results were collected from duplicated experiments and data were presented as mean ± SD. Comparisons among groups were made using one-way anova test with Dunnett's post hoc test analysis. Statistical analysis was performed with Prism 5 software (GraphPad Software, Inc, La Jolla, CA, USA) and *P*-value less than 0.05 was considered as statistically significant. *P < 0.05; **P < 0.01 and ***P < 0.005 represent significant difference compared with control group; ^+^*P* < 0.05 and ^++^*P* < 0.01 represent significant difference compared with CY group.

## Results

### The spleen mass was reduced by Rg1 treatment

**Figure 1:**
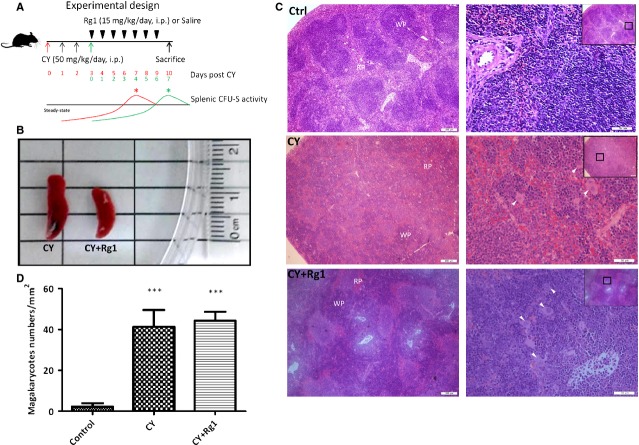
The gloss and histological examination of the spleen after ginsenoside Rg1 treatment in CY-treated mice. (**A**) The schema in experimental design. On day of killing (equal to 7–10 days after CY), mice that received CY should have the highest EMH activity in the spleen without Rg1 treatment. The curve for CFU-S activity in the spleen of mice after CY treatment was referred to the published data from Šefc *et al*. 10. (**B**) The spleen size was significantly decreased after Rg1 treatment in CY-treated mice. (**C**) Histological sections of the spleen at the magnifications showed that the borders between the WP and RP under normal conditions were vanished and replaced by a diffused form of pulps without any distinctive margin in CY-treated mice. The hyperplasia of megakaryocytes within the WP of the spleen was also predominant. After Rg1 treatment, the architecture of the spleen was reorganized with a mixture of nodular and diffused pulps. However, the megakaryocytes still prevailed. (**D**) Average number of megakaryocytes in 400× field of magnification (equal to 0.38 mm^2^ in a calculated area). Scale bars = 200 μm (left) and 50 μm (right). ***P < 0.005 compared with control group. Abbreviations: CY: cyclophosphamide; WP: white pulp; RP: red pulp; EMH: extramedullary haematopoiesis.

Cyclophosphamide was reported to induce myelosuppression, mobilization of HSCs and EMH in the spleen 10,19, especially the highest EMH activity in the spleen was detected on day 7 after CY treatment 10. Cyclophosphamide treatment was regularly accompanied by splenomegaly. Previous report from Xu *et al*. indicated that Rg1 treatment could improve the frequency of LSK HSPCs and the haematopoietic function in the bone marrow of CY-treated mice 17. These results prompted us to examine a possible effect of Rg1 on alleviation of EMH in the spleen induced by CY. According to clinical usage, we administrated a single dose of CY (50 mg/kg) once a day for four consecutive days and a total dose was up to 200 mg/kg for a mouse. Ginsenoside Rg1 treatment followed for next 7 days by a dose of 15 mg/kg/day (Fig.1A). We killed the mice on day 10 when EMH activity was the most active in the spleen after CY treatment 10. As the results, we had shown that the spleen size was reduced after Rg1 treatment (Fig.1B). We further calculated the cellularity of the spleen, and total cell number of the spleen was significantly decreased after Rg1 treatment (Table1). Moreover, both weight of the spleen and spleen to body weight ratio were significantly reduced in CY plus Rg1 group compared to CY group (Table1). Histological examination showed solid and well demarcated margin between the red and white pulp in the spleen of the control mice (Fig.1C); but after treatment of CY, the pulps became disorganized and the interspace between red and white pulp was enlarged. Erythropoiesis and granulopoiesis also prevailed in the interspaces. Megakaryocytes were frequently found in the spleen after CY treatment providing a strong evidence of EMH. In CY-treated mice further receiving Rg1 treatments, pulps mixed with nodular and diffused types appeared in the spleen indicating a reorganizing trend. However, megakaryocytes were still present in the spleen of Rg1-treated mice compared to that in control mice (44.2 ± 4.5 *versus* 2.3 ± 1.7/mm^2^, *P* < 0.05) (Fig.1D), indicating that EMH was not actually eradicated by Rg1 treatment. Notably, the bone marrow cellularity in all three groups was similar at time of the examination.

**Table 1:** The characteristics of the spleen in mice among the experimental groups

Group	Cell counts (×10^7^)		Spleen weight (g)	Spleen dimension (mm^3^)	Spleen-body weight ratio
	BM	Spleen			
Control	3.03 ± 0.3	5.89 ± 0.6	0.06 ± 0.002	n.a	0.003 ± 0.00008
CY	3.16 ± 0.4	10.37 ± 2.5*	0.18 ± 0.003***	66.7 ± 8.3	0.010 ± 0.0008***
CY+Rg1	3.09 ± 0.4	4.47 ± 0.8†	0.10 ± 0.01**,††	46.7 ± 3.5	0.005 ± 0.008*,††

^*^P < 0.05; ^*^^*^P < 0.01; ^*^^*^^*^P < 0.001 compared with control group; ^†^*P* < 0.05; ^††^*P* < 0.001 compared with CY group.

BM: bone marrow; CY: cyclophosphamide; Rg1: ginsenoside Rg1.

### The absolute cell number of c-Kit^+^ HSPCs in the spleen was decreased after Rg1 treatment in CY-treated mice

**Figure 2:**
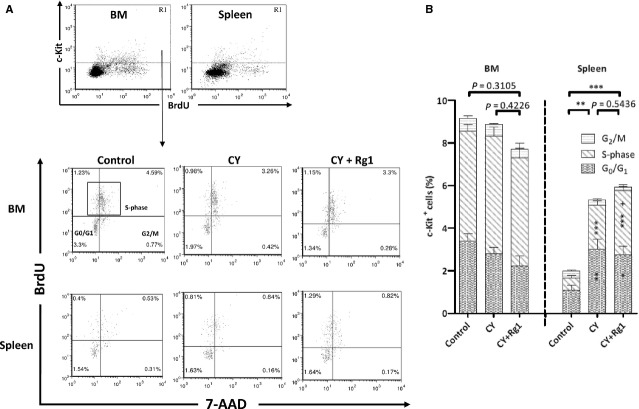
Rg1 treatment increased the proliferation activity of c-Kit^+^ haematopoietic stem/progenitor cells in the spleen of CY-treated mice. (**A**) Fluorescence-activated cell sorting (FACS) analysis of c-Kit positive cells (R1) and the fraction of c-Kit^+^ cells in S-phase (cubic box) in the bone marrow and the spleen. (**B**) After Rg1 treatment, the fraction of c-Kit^+^ cells in S-phase was significantly increased in the spleen. The fractions of c-Kit^+^ HSPCs in S-phase in bone marrow among three groups were showing no significant difference. In the spleen, the frequency of c-Kit^+^ cells in CY and CY plus Rg1 group were significantly higher compared to that in control group, indicating EMH of the spleen. *P < 0.05, **P < 0.01 and ***P < 0.005 compared with control group; ^+^*P* < 0.05 compared with CY group. Results were the mean ± SEM,* n* = 5, in a representative experiment. Abbreviations: CY: cyclophosphamide; BrdU: 5-bromo-2′-deoxyuridine; 7-AAD: 7-amino-actinomycin D; HSPCs: haematopoietic stem and progenitor cells; EMH: extramedullary haematopoiesis.

The activity of EMH in the spleen was also determined by the presence of c-Kit^+^ HSPCs using flow cytometry analysis (Fig.2A). As expected, the frequency of c-Kit^+^ HSPCs in the spleen was significantly increased in mice treated with CY showing EMH (2.0 ± 0.4 *versus* 5.3 ± 0.7, *P* < 0.005). However, mice further receiving Rg1 treatment did not alter the frequency of c-Kit^+^ HSPCs in the spleen. This again indicated that EMH in the spleen of CY-treated mice was not eradicated by Rg1 treatment. However, as a result of a significant decrease in cellularity of the spleen after Rg1 treatment in CY-treated mice, an absolute number of c-Kit^+^ HSPCs was significantly decreased in the spleen (Table2). Hence, these results demonstrated that Rg1 treatment could alleviate EMH of the spleen induced by CY. In contrast, the frequency of c-Kit^+^ HSPCs in bone marrow was not affected by Rg1 treatment (Fig.2B).

**Table 2:** The cell count and frequency of proliferating HSPCs in BM and the spleen

	LSK cell counts (×10^4^)	c-Kit^+^ cells counts (×10^6^)	Counts of c-Kit^+^ cell in S-phase (×10^5^)	Frequency of c-Kit^+^ cells in S-phase of all cells (%)	Proliferation activity of total c-Kit^+^ cells (%)
BM					
Control	4.4 ± 1.2	2.8 ± 0.6	15.7 ± 0.9	5.2 ± 0.3	56.8 ± 2.0
CY	5.1 ± 1.0	2.8 ± 0.5	17.5 ± 1.4	5.5 ± 0.4	62.2 ± 1.4
CY+Rg1	11.7 ± 1.3**,††	2.4 ± 0.8	15.7 ± 2.1	5.1 ± 0.7	65.2 ± 3.1*
Spleen					
Control	0.02 ± 0.01	1.2 ± 0.5	3.3 ± 0.7	0.57 ± 0.12	28.3 ± 0.6
CY	1.8 ± 0.4***	5.5 ± 1.7***	18.6 ± 1.9***	2.07 ± 0.20***	39.6 ± 1.5**
CY+Rg1	0.6 ± 0.2††	2.6 ± 0.6††	13.4 ± 1.6***,†	3.02 ± 0.27***,†	51.5 ± 3.2***,††

^*^P < 0.05; ^*^^*^P < 0.01, ^*^^*^^*^P < 0.001 compared with control group; ^†^*P* < 0.05, ^††^*P* < 0.001 compared with CY group.

Proliferation activity of total c-Kit^+^ cells (%) = (the number of c-Kit^+^ cells in S-phase/total c-Kit^+^ cells) × 100, calculated by flow cytometry software (BD FACSDiva) post-analysis.

BM: bone marrow; CY: cyclophosphamide; Rg1: ginsenoside Rg1; LSK: lineage negative, Sca-1 positive and c-Kit positive; HSPCs: haematopoietic stem and progenitor cells.

### Rg1 treatment did not suppress the apoptosis of c-Kit^+^ HSPCs induced by CY

**Figure 3:**
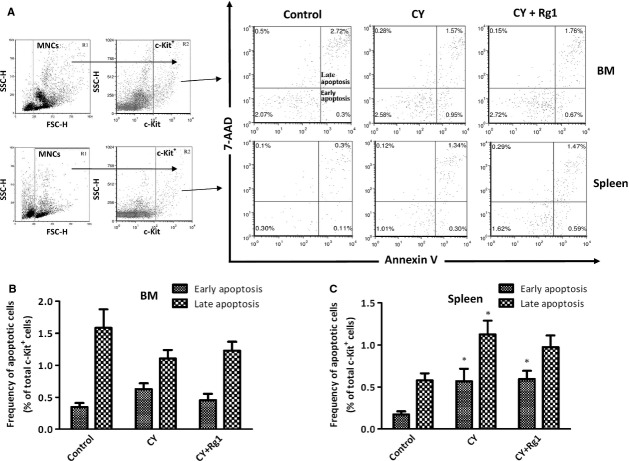
Rg1 treatment did not alter apoptosis for c-Kit^+^ HSPCs in CY-treated mice. (**A**) FACS analysis of early- (Annexin V^+^/7-AAD^−^) and late- apoptotic cells (Annexin V^+^/7-AAD^+^) in the fraction of c-Kit^+^ cells (R2) in bone marrow and the spleen. (**B** and **C**) Rg1 treatment did not cause cell death for c-Kit^+^ HSPCs from early- and late- apoptosis in the bone marrow and the spleen of CY-treated mice. Results were the mean ± SEM,* n* = 5, in a representative experiment. *P < 0.05 compared with control group. Abbreviations: CY: cyclophosphamide; HSPCs: haematopoietic stem and progenitor cells.

To test a possible effect of Rg1 on the protection of HSPCs from apoptosis, we used Annexin V and 7-AAD to discriminate early- and late-apoptotic cells in the c-Kit^+^ fraction of bone marrow and in the spleen (Fig.3A). There was an increased fraction of apoptotic c-Kit^+^ HSPCs in the spleen in mice treated with CY (Fig.6C). However, there were no significant differences between CY and CY plus Rg1 groups, indicating that Rg1 was neither involved in protecting nor causing apoptosis for c-Kit^+^ HSPCs (Fig.3B and C). It also demonstrated that the reduced absolute number of c-Kit^+^ HSPCs in the spleen was not caused by increased cell death.

### Ginsenoside Rg1 increased proliferation activity of c-Kit^+^ HSPC in the spleen of CY-treated mice

The proliferation activity of c-Kit^+^ HSPCs was analysed by flow cytometry. Interestingly, the frequency of c-Kit^+^ HSPCs in S-phase was not affected by Rg1 treatment in the bone marrow of CY-treated mice (Fig.2B). In contrast, frequency of c-Kit^+^ HSPCs in S-phase was increased in the spleen after Rg1 treatment (2.1 ± 0.2% *versus* 3.0 ± 0.3%, *P* < 0.05) and an increase in proliferation activity of total c-Kit^+^ cells (39.6 ± 1.5% *versus* 51.5 ± 3.2%, *P* < 0.05) in the spleen after Rg1 treatment in CY-treated mice (Table2). Because of the reduced cellularity of the spleen in CY plus Rg1-treated mice, absolute number of c-Kit^+^ HSPCs in S-phase was remarkably decreased (18.6 ± 1.9 × 10^5^ *versus* 13.4 ± 1.6 × 10^5^, *P* < 0.05). We suggested that a fraction of proliferating c-Kit^+^ HSPCs was mobilized from the spleen into the peripheral blood.

### Rg1 increased c-Kit^+^ HSPCs circulating in blood only in presence of EMH in the spleen

**Figure 4:**
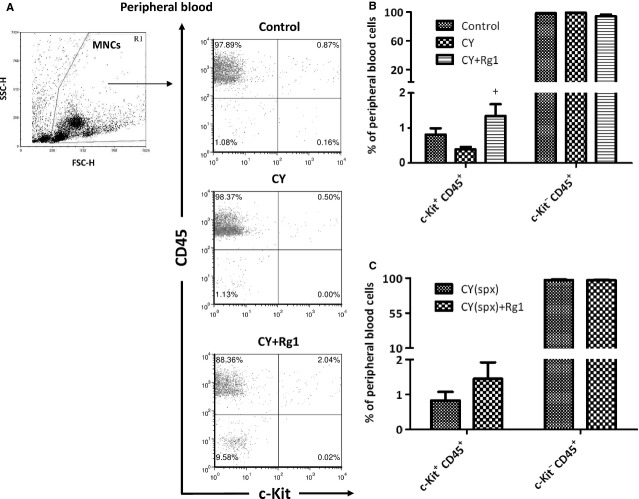
Rg1 treatment increased the percentage of c-Kit^+^ haematopoietic stem/progenitor cells in the peripheral blood of CY-treated mice upon EMH of the spleen. (**A**) FACS analysis of c-Kit^+^/CD45^+^ HSPCs and c-Kit^−^/CD45^+^ mature blood cells in the peripheral blood. (**B**) The frequency of c-Kit^+^/CD45^+^ cells was significantly increased in the peripheral blood after Rg1 treatment in CY-treated mice. However, the frequency of c-Kit^−^/CD45^+^ mature blood cells was unaffected after Rg1 treatment. (**C**) The frequency of c-Kit^+^/CD45^+^ cells was no longer increased after Rg1 treatment in CY-treated splenectomized mice. ^+^*P* < 0.05 compared with CY group. Results were the mean ± SEM,* n* = 5, in a representative experiment. Abbreviations: CY: cyclophosphamide; spx: splenectomy; MNCs: mononuclear cells; FSC-H: forward scatter-height; SSC-H: side scatter-height; HSPCs: haematopoietic stem and progenitor cells; EMH: extramedullary haematopoiesis.

To confirm the mobilization of HSPCs from the spleen, we examined c-Kit^+^ HSPCs in the peripheral blood (Fig.4). After Rg1 treatment, the frequency of HSPCs (c-Kit^+^/CD45^+^ cells) was significantly increased in the peripheral blood of CY-treated mice (0.4 ± 0.07% *versus* 1.3 ± 0.3%, *P* < 0.05). In contrast, mature blood cells (c-Kit^−^/CD45^+^ cells) were not affected by Rg1 treatment (Fig.4B). Interestingly, frequency of c-Kit^+^/CD45^+^ HSPCs in peripheral blood was not increased in CY-treated splenectomized mice after Rg1 treatment (Fig.4C). In concert with our results, Xu *et al*. had also shown that Rg1 treatment increased LSK HSPCs in the peripheral blood in CY-treated mice 17. Our results strongly suggested that HSPCs were mobilized from the spleen into the blood after Rg1 treatment in CY-treated mice.

### Rg1 treatment decreased number of LSK HSPCs in the spleen in CY-treated mice

**Figure 5:**
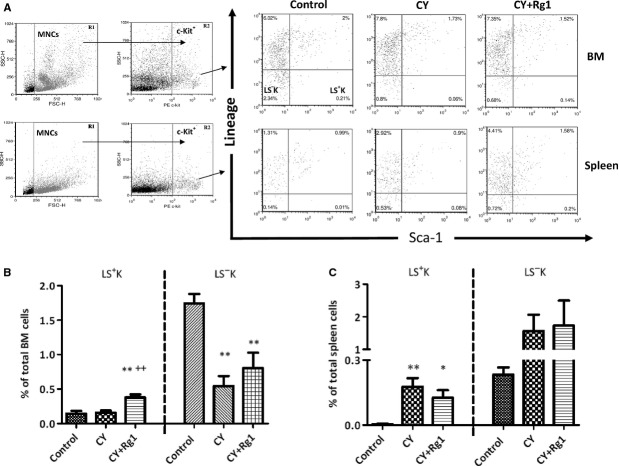
Rg1 treatment increased lineage (Lin^−^) Sca-1^+^ c-Kit^+^ (LSK) fraction in the bone marrow, but not in the spleen of CY-treated mice. (**A**) FACS analysis of LSK haematopoietic stem/progenitor cells and Lin^−^, Sca-1^−^ c-Kit^+^ (LS^−^K) progenitors in the bone marrow and the spleen. (**B**) The frequency of LSK cells was increased in bone marrow of CY-treated mice after Rg1 treatment. Moreover, the frequency of LS^−^K in CY and CY plus Rg1 groups were lower compared to that in control group, indicating myelosuppression in bone marrow induced by CY. (**C**) There were no significant differences in both LSK and LS^−^K fractions in the spleen after Rg1 treatment in CY-treated mice. In addition, the frequency of LSK in the spleen in CY and CY plus Rg1 groups were higher compared to that in control group, indicating EMH of spleen. *P < 0.05 and **P < 0.01compared with control group; ^++^*P* < 0.01 compared with CY group. Results were the mean ± SEM,* n* = 5, in a representative experiment. Abbreviations: CY: cyclophosphamide; MNCs: mononuclear cells; FSC-H: forward scatter-height; SSC-H: side scatter-height; PE: phycoerythrin; EMH: extramedullary haematopoiesis.

A previous report from Xu *et al*. indicated that the frequency of LSK HSCs was significantly increased both in the bone marrow and in the peripheral blood of CY-treated mice after Rg1 treatment 17. We confirmed the increase in bone marrow LSK cells induced by Rg1 treatment in CY-treated mice (0.16 ± 0.03% *versus* 0.38 ± 0.04%, *P* < 0.01; Fig.5B) and showed that another subset of Lin^−^ c-Kit^+^ cells, that of Lin^−^, Sca-1^−^ c-Kit^+^ (LS^−^K) cells was unaffected (Fig.5B). However, the LS^−^K cells were significantly decreased in bone marrow from CY or CY plus Rg1-treated mice compared to control mice (Fig.5B) as a result of the myelosuppression induced by CY. In the spleen, although the frequencies of both LSK and LS^−^K HSPCs were unaffected after Rg1 treatment, LSK cell number was actually significantly decreased because of significant reduction in splenic cellularity (Table2). Nonetheless, the frequency of LSK HSPCs in the spleen was still significantly higher in CY plus Rg1 mice compared to that in control mice (0.004 ± 0.005% *versus* 0.13 ± 0.07%, *P* < 0.05), showing that EMH was still present in a diminished extend in the spleen after Rg1 treatment (Fig.5C).

### The effect of Rg1 on bone marrow was suppressed in splenectomized mice

**Figure 6:**
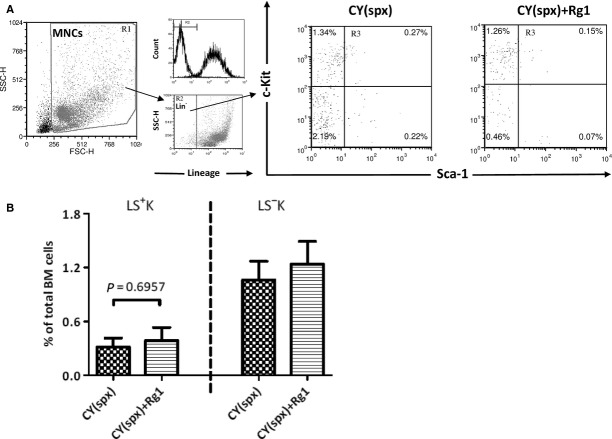
Rg1 treatment was unable to increase LSK fraction in the bone marrow in CY-treated splenectomized mice. (**A**) FACS analysis of LSK cells in the bone marrow (R3). (**B**) LSK fraction was unable to be increased in the bone marrow after Rg1 treatment in CY-treated mice that prior received splenectomy. **P < 0.01 compared with control group; ^++^*P* < 0.01 compared with CY group. Results were the mean ± SEM,* n* = 5, in a representative experiment. Abbreviations: CY: cyclophosphamide; spx: splenectomy; MNCs: mononuclear cells; FSC-H: forward scatter-height; SSC-H: side scatter-height.

When we examined the effect of Rg1 at LSK HSPCs cell numbers in bone marrow using splenectomized mice (Fig.6A), there was no longer a significant increase (Fig.6B). Rg1 thus improved LSK HSPCs cell numbers in bone marrow only in the presence of EMH in the spleen.

## Discussion

Haematopoiesis is a hierarchical cell system aimed at the production of all types of blood cells in desired quantities 20. The system depends on the presence of stem and progenitor cells which not only maintain a continuous production of blood cells but also allow for a rapid regeneration of the haematopoietic tissue in case of its damage or after transplantation 21. There are several reports which show that spleen participates in the bone marrow haematopoietic reconstitution in irradiated mice after transplantation 22,23. Filip *et al*. observed a significant reduction in HSPCs count in the bone marrow of splenectomized mice that received irradiation and transplantation compared to sham-operated control mice 22. It is also well known that transplanted murine HSPCs have the capacity to form macroscopic colonies in the spleens of irradiated mice 24. Similarly, human foetal liver cells are able to form spleen colonies consisting of haematopoietic tissue when injected into lethally irradiated mice 25. This shows that the splenic microenvironment of mice can adopt HSPCs derived from both the mouse and human haematopoietic tissue. Besides, the spleen is an active haematopoietic organ in adult mice though HSCs are approximately 10 times less frequent than in bone marrow. In a parabiosis model, only 9% of HSCs (CD34^−^ LSK cells) exchange in the partner spleens despite intensive blood flow through the spleen 26. Furthermore, these endogenous HSCs in the spleen also gave rise to long-term reconstitution upon adoptive transfer into irradiated recipient mice. Regarding cell cycle activity, the CD34^−^ LSK cells were two times more actively proliferating in the spleen compared to those in the bone marrow in adult mice 26. In reconstituted mice, donor bone marrow CD34^−^ LSK cells were also found in cycle more frequently in the spleen suggesting an organ-specific regulation for HSCs cycling 26. It could be suggested that HSCs initiate an expansion first in the spleen after their transplantation prior to homing to the bone marrow. However, this hypothesis is controversial as the human spleen does not support haematopoiesis postnatally, except in case of EMH 27.

Cyclophosphamide is one of the most common drugs used in cancer chemotherapy and for patient conditioning prior to HSC transplantation 10. A patient who received four cycles of chemotherapeutic agents including CY for treatment of lymphoma presented with splenomegaly. The spleen sections showed EMH without lymphomatous involvement 9. In mice, treatment of CY causes cytotoxicity to macrophages and osteoblasts in the bone marrow that results in a decreased expression of cytokines and vascular cell adhesion molecule 1 (VCAM-1), respectively, and impairs mineralization and growth of the endosteum 19,28. Cyclophosphamide also stimulates a stepwise accumulation of matrix metallopeptidase 9 (MMP-9) in extracellular fluid that eventually disrupts the CXCR4/CXCL12 chemotactic interaction between HSPCs and stromal cells in the bone marrow 29. Therefore, CY causes myelosuppression and mobilizes HSPCs into the blood. A part of the action of CY is indirect because of the short half-life of its cytotoxic metabolite, phosphoramide mustard 30. In mice, a single high dose of CY induces intensive myelopoiesis in the spleen 10. Flow cytometry analysis also demonstrated an increase in HSPCs expressing CD34^+^ or CD117^+^ (c-Kit) in the spleen on day 7 after CY treatment 11. Haematopoiesis in the spleen transiently represented about 70% of the total body haematopoiesis at the peak of the EMH expansion 31. All these indicated that spleen became a major reservoir of HSPCs in response to CY treatment within a week 10,11. Extramedullary haematopoiesis in the spleen converts spleen from a predominant lymphoid tissue to a tissue with profound myelopoiesis 10,32. Wang *et al*. observed that the levels of WBCs, platelets, RBCs and haemoglobin did not recover in splenectomized mice to the levels of normal control mice by 21 days after CY injection. This result indicated that spleen may help animals to restore haematopoiesis after CY 11. Furthermore, the cell cycle activity of HSCs in the spleen was much higher than that in bone marrow between days 5 and 7 after CY administration 10. Hence, recovery of bone marrow myelopoiesis after CY treatment might be through the induction of EMH in the spleen.

Several reports have shown that EMH in the spleen could be relieved when the growth of malignant HSCs in the bone marrow was suppressed by innovative drugs 33,34. In our study, we suggested that while the bone marrow activity in CY-treated mice was ameliorated by Rg1 treatment, splenic EMH could also be alleviated. However, histological examination of spleens of CY and Rg1-treated mice revealed that megakaryocytes were still present in high numbers, indicating that Rg1 was unable to eliminate EMH of the spleen induced by CY.

The receptor tyrosine kinase c-Kit is critical for their proliferation, survival and differentiation of HSPCs 35. CFU-S activity was absent in lineage^−^ c-Kit^−^ fraction of bone marrow cells 36. Therefore, we employed flow cytometry to examine the fraction of c-Kit^+^ HSPCs in both the bone marrow and spleen. We found that the frequency of c-Kit^+^ HSPCs as well as their proliferation activity was not influenced in bone marrow by Rg1 treatment in CY-treated mice. On the contrary, the proliferation activity of c-Kit^+^ HSPCs in the spleen was increased after Rg1 treatment (Fig.2B and Table2) but the absolute number of c-Kit^+^ HSPCs was significantly decreased (Table2). Interestingly, c-Kit^+^ HSPCs apoptosis was unaffected after Rg1 treatment in the spleen of CY-treated mice, ruling out the possibility of cell death of c-Kit^+^ HSPCs induced by Rg1. Therefore, the significant reduction in c-Kit^+^ HSPCs in the spleen after Rg1 treatment reflected the mobilization of HSPCs from the spleen. We also detected an increased population of c-Kit^+^/CD45^+^ HSPCs in the peripheral blood (Fig.4B) confirming previous observation of Xu *et al*. 17. We strongly suspected that elevated c-Kit^+^/CD45^+^ HSPCs in blood after Rg1 treatment were mobilized from the spleen. We confirmed this hypothesis using splenectomized mice which did not exhibit increased c-Kit^+^ HSPCs numbers in the peripheral blood after Rg1 treatment (Fig.3B). Hence, Rg1 treatment in CY-treated mice can promote proliferation as well as mobilization of c-Kit^+^ HSPCs in the spleen.

As in the study of Xu *et al*. 17, Rg1 treatment increase bone marrow LSK HSPCs in CY-treated mice (Fig.5B). Interestingly, we noted that LSK HSPCs were not increased in CY and Rg1-treated splenectomized mice. This demonstrated that the effect of Rg1 on increasing bone marrow LSK cells depend on the presence of EMH in the spleen, rather than by directly promoting the expansion of LSK cells in bone marrow. In concert with this, Xu *et al*. demonstrated that the treatments with Rg1 did not increase bone marrow LSK cells in normal mice lacking EMH.

In a recent study, ginsenoside Rg1 promoted proliferation of bone marrow-derived stromal cells (BMSCs) *in vitro* through an oestrogen receptor-mediated response 37. Jin-Xiang *et al*. indicated that BMSCs stimulated with recombinant human macrophage colony-stimulating factor (rhM-CSF) have a high seeding efficiency to bone marrow while injected intravenously to severe combined immunodeficiency (SCID) mice. Moreover, the co-transplantation of rhM-CSF-stimulated BMSCs with HSCs facilitated haematopoietic reconstitution in irradiated mice 38. According to previous reports, ginsenoside Rg1 may increase the macrophage colony-stimulating factor (M-CSF) mRNA level and protein production in osteoblasts through the enhancement of the expression of IL-4 in CD4^+^ T cells 39,40. Therefore, ginsenoside Rg1 treatment to mice given CY might promote the migration of splenic HSPCs to bone marrow by increasing the proliferation of BMSCs and M-CSF up-regulation in the bone marrow.

Ginsenoside Rg1 can also stimulate production of VEGF and promote endothelial progenitor cell (EPC) proliferation 41,42. Several reports indicated that P- and E-selectin expressed on EPC were critical for the post-transplantation homing of HSCs to bone marrow 43,44. Furthermore, oestrogens increase the expression of E-selectin, intercellular adhesion molecule-1 and VCAM-1 in endothelial cells 45 and ginsenoside Rg1 was shown to interact with the oestrogen-response-element in HeLa cells 46. We predict that ginsenoside Rg1 may enhance homing of splenic HSPCs to bone marrow through increasing the expression of E-selectin and the adhesion molecules. In future, we will test the possible effect of Rg1 at the expression level of E-selectin and the adhesion molecules on EPC.

Until now, EMH was viewed largely only as a compensatory response to the insufficient production of blood cells by bone marrow 47–49. Our study on Rg1 provides a paradigm to demonstrate the beneficial action of splenic EMH on the bone marrow haematopoietic activity in myelosuppressed mice, an action of EMH which exceeds its compensatory role.
